# Complete mitochondrial DNA sequence of the giant water bug *Kirkaldyia deyrolli* (Hemiptera: Belostomatidae).

**DOI:** 10.1080/23802359.2020.1833774

**Published:** 2020-11-11

**Authors:** Juiki Nakasako, Hisashi Okuyama, Shinya Ohba, Jun-ichi Takahashi

**Affiliations:** a Faculty of Life Sciences, Kyoto Sangyo University, Kyoto, Japan; b Biological Laboratory, Faculty of Education, Nagasaki University, Nagasaki, Japan

**Keywords:** Next generation sequencing, giant water bug, *Kirkaldyia deyrolli*, endangered species

## Abstract

Giant water bugs (genus *Kirkaldyia* and *Lethocerus*) are well known species from aquatic habitats throughout the world’s subtropical and tropical areas. Only one species of the *Kirkaldyia deyrolli* is distributed in Japan. *K. deyrolli* has been designated as second category rare species according Law for Conservation of Endangered Species of Wild Fauna and Flora in Japan, in 2019. We analyzed, for the first time, the complete mitochondrial genomes of the giant water bug *K. deyrolli* from Japan using next-generation sequencing. The mitochondrial genome was a circular and 15,579 bp molecule that included 13 protein-coding genes (PCGs), 22 tRNA genes, and two ribosomal RNA genes, along with one AT-rich control region. The AT content value was 69.55%. The heavy strand was predicted to have nine PCGs and 15 tRNA genes, whereas the light strand was predicted to contain four PCGs, seven tRNA genes, and two rRNA genes. Start codons were variable for all PCGs: four ATA, three ATC, four ATG, two ATT genes as the start codon. Stop codons were of two types: TAA for 12 genes and TAG for one gene. Incomplete stop codon T was identified. The molecular phylogenetic relationship, inferred using 13 PCGs, was consistent with that reported in previous studies, which predicted a sister relationship to the genus *Lethocerus*.

*Kirkaldyia deyrolli* (Vuillefroy, 1864) distributes Southeastern Asia, China, Taiwan, South Korea, and Japan. Before a paper published by Goodwyn (2006) *K. deyrolli* was known as *Lethocerus deyrollei* or *L. deyrolli*. Abundance of *K. deyrolli* has decreased rapidly during last five decades (Ohba 2011; Ohba and Nakasuji 2006), and they are designated as threatened-vulnerable species in the Japanese Red List (Ministry of the Environment 2015). Nevertheless, few studies have focused on the phylogenetic relationship of Belostomatidae and the phylogenetic relationships between *K. deyrolli* populations also are not fully understood.

We collected adult *K. deyrolli* on pond in Hitoyoshi City, Kumamoto Prefecture, Japan, in 2019 (32°13′N, 130°46′E). Sample was transferred immediately to 99.5% ethanol for mitochondrial DNA analysis. Total DNA was extracted from one adult using the DNeasy mini kit (QIAGEN, Hilden, Germany). The gDNA library used for sequencing was prepared using the KAPA Hyper Prep kit, and a MiSeq sequencer (ILLUMINA, United States) was used to sequence the whole genome with an Illumina reagent kit. The gDNA library was indexed and run simultaneously over 600 cycles yielding paired reads of 250 bp. The complete mitochondrial genome of the Korean *K deyrolli* was used as a reference sequence (KU237288). The resultant reads were assembled and annotated using the Glimmer program of Geneious R9 software (Kearse et al. 2012) and the MITOS web server (Bernt et al. 2013). Thirteen protein-coding genes (PCGs) and two rRNA genes sequences were aligned using Genetyx version 15 (GENEYTX, Japan). The phylogenetic analysis (Maximum Likelihood analysis) was based on the nucleotide sequences of 13 protein-coding genes using MEGA X (Kumar et al. 2018). The T92 + G model was selected from the find best DNA program in MEGA X.

We succeeded in sequencing the entire mitochondrial genome of *K. deyrolli* from Japan (DDBJ accession number LC567844). This specimen stored at the National Museum of Nature and Science, Japan (sample number NSMT-I-He 83310). The genome consisted of a closed loop 15,579 bp long and included 13 PCGs, 22 tRNA genes, two rRNA genes, and one AT-rich control region that represented a typical Hemipteran mitochondrial genome (Devi et al. 2016). The heavy strand was predicted to have 12 PCGs and 14 tRNA, while the light strand was predicted to contain one PCG, eight tRNA genes, and two rRNA genes. Start codons were variable for all PCGs: four genes used ATA; and *ATP8*, *ND1* and *ND6* used ATC; and *COIII*, *ATP6*, *ND4* and *Cytb* used ATG; and *ND4L* and *ND5* used ATT as the start codon. Stop codons were of two types: TAA for 12 genes and TAG for one gene. Incomplete stop codon T (*COII* and *ND3*) was identified. The genes *ATP8* and *ATP6* shared 10 nucleotides, and *ATP6* and *COIII* shared one nucleotide. The genes *ND4* and *ND4L*, and genes *ND5* and *ND6* shared 7 and 4 nucleotides, respectively. Molecular phylogenetic analysis revealed that *K. deyrolli* in Japan was genetically differentiated enough to be considered a unique population distinct from the *K. deyrolli* from South Korea and China (Sareein et al. 2019).

## Data Availability

The data that support the findings of this study is openly available in NCBI at https://www.ncbi.nlm.nih.gov/sra?term=DRA010833, accession number DRA010833 and DDBJ at http://getentry.ddbj.nig.ac.jp/getentry/na/LC567844./, accession number LC567844. The molecular phylogenetic tree (maximum likelihood analysis) of the Belostomatidae based on the nucleotide sequences of the 13 protein-coding genes of the mitochondrial genome. Sequences from *Diplonychus esakii* (MK251109) was used as an outgroup. These sequences were separated by codon positions, and for each partition, the optimal models of sequence evolution were used in the MEGA X, based on the corrected Akaike information criterion. The numbers at the nodes indicate the bootstrap support inferred from 1000 bootstrap replicates. Alphanumeric terms indicate the DNA Database of Japan accession numbers..
